# Leptin Increases Blood Pressure and Markers of Endothelial Activation during Pregnancy in Rats

**DOI:** 10.1155/2013/298401

**Published:** 2013-09-19

**Authors:** Hisham Saleh Ibrahim, Effat Omar, Gabrielle Ruth Anisah Froemming, Harbindar Jeet Singh

**Affiliations:** Faculty of Medicine, Universiti Teknologi MARA, 47100 Sg Buloh, Selangor, Malaysia

## Abstract

Raised leptin levels have been reported in the placentae and serum of women with elevated blood pressure and proteinuria during pregnancy. The role of leptin in this however remains unknown. This study investigates the effect of leptin administration on systolic blood pressure (SBP) and proteinuria and serum markers of endothelial activation during pregnancy in *Sprague Dawley rats*. From day 1 of pregnancy, 24 rats were randomised into those given either saline (group 1) or leptin at 60 or 120 **μ**g/kg/body weight/day (groups 2 and 3 resp.). SBP was measured every 5 days and 24-h urinary protein was measured at days 0 and 20 of pregnancy. Animals were euthanised on day 20 of pregnancy, and serum was collected for estimation of E-selectin and ICAM-1. Compared to group 1, SBP during the latter part of the pregnancy was significantly higher in the leptin-treated group (*P* < 0.01). Urinary protein excretion, serum E-selectin, and ICAM-1 were significantly higher in leptin-treated rats (*P* < 0.05). It seems that leptin administration to normotensive *Sprague Dawley rats* during pregnancy significantly increases SBP, urinary protein excretion, and markers of endothelial activation. However, further studies are required to examine the underlying mechanism responsible for this and its relevance to preeclampsia in humans.

## 1. Introduction

Leptin, a 16 kDa protein, is primarily secreted from white adipose tissue and plays an important role in controlling food intake and energy balance [1, 2]. Exogenous leptin treatment decreases adiposity by decreasing food intake and increasing energy utilization [3–5]. In addition to suppressing appetite, leptin also promotes weight loss in rodents via sympathetic activation of brown adipose tissue and oxidation of fatty acids [6]. Sympatho-activation following either intra-cerebroventricular or chronic intravenous leptin administration has been shown to increase mean arterial blood pressure and heart rate in rodents [7–9]. Arterial blood pressure in leptin-deficient *ob/ob* mice is lower than in wild-type littermates and is substantially increased with exogenous leptin administration [10]. Agouti mice, which are obese and hyperleptinemic, and transgenic skinny hepatic leptin over-expressing mice have elevated blood pressure [10]. Taken together, these observations demonstrate in several different rodent models that leptin influences blood pressure and that hyperleptinemia in obesity might contribute to the hypertension.

Studies on nonpregnant obese individuals also indicate that adipose tissue expresses high levels of proinflammatory cytokines [11, 12], and the proinflammatory state of obesity has been implicated in pre-eclampsia [13]. Maternal obesity in pregnancy is associated with increases in serum leptin, MCP-1, and hsCRP [14]. Increased leptin concentrations have been found to correlate with increased concentration of inflammatory markers in morbidly obese individuals [15]. Elevated serum leptin levels have been reported in women with preeclampsia [16]. Leptin concentrations have also been found to be higher in the placentae of preeclamptic women [17]. In addition to raised serum leptin level, generalised endothelial dysfunction is evidently also present in women with pre-eclampsia [18, 19]. The cause for this widespread endothelial dysfunction is unknown, although numerous placental factors have been proposed. As the prevalence of pre-eclampsia is higher in obese women and elevated serum leptin concentrations therefore precede pre-eclampsia [16], we wondered if the raised serum leptin concentration contributed to the hypertension, proteinuria, and endothelial dysfunction that are commonly seen in women with hypertensive disorders of pregnancy. This study therefore examined the effects of exogenous leptin administration during pregnancy on blood pressure, urinary protein excretion, markers of endothelial activation, and food and water intake in non-obese normotensive *Sprague Dawley rats*.

## 2. Methods

Female *Sprague Dawley rats*, aged 12-13 weeks, were housed in polyethylene cages at room temperature (22–24°C), with a 12/12 hours light/dark cycle (7am–7pm), and with access *ad libitum to *food and water throughout the observation period. All animals were screened for raising blood pressure and proteinuria before the start of the study. Animals with systolic blood pressure (SBP) of ≥140 mm Hg and/or positive for proteinuria of (+) on the dipstick test were excluded from the study. After confirmation of proestrous following a vaginal smear, each female was housed overnight with a fertile male. Mating was confirmed through a positive sperm smear the following morning. This was marked as pregnancy day 1. Following confirmation of mating, the females were randomised into 3 groups, with a total of 8 rats per group (Table 1). Group 1 (NSP) was given 0.1 mL saline subcutaneously from day 1 of pregnancy; group 2 (LD1-60) was given 60 *μ*g/kg/day of leptin subcutaneously from day 1 of pregnancy, and group 3 (LD1-120) was given 120 *μ*g/kg/day of leptin subcutaneously from the day 1 of pregnancy. The dose of leptin used was based on our previous study in the male rat where 30 *μ*g/kg/day was found to significantly affect sperm count and morphology without significantly affecting food or water intake [20]. The initial starting dose was doubled to 60 *μ*g/kg/day in this study in view of the slight leptin resistance that is usually present during pregnancy. The dose of leptin was adjusted according to the change in body weight as the pregnancy progressed.

Body weight and food and water intake were recorded every 7 days, while 24-hour urine specimen for measurement of protein excretion was collected on days 0 and 20 of pregnancy. SBP was measured every 5 days during pregnancy using tail-cuff plethysmography (CODA, USA). All animals were decapitated using a small animal guillotine on day 20 of pregnancy, and blood samples were then collected for the analysis of ICAM-1 and E-selectin. Serum ICAM-1 was measured using a commercially available kit (Abnova, USA) as described in a previous study [21]. The serum E-selection was measured using a commercially available kit (*IBL *JAPAN) after Vadasz et al. [22]. Urinary protein excretion was estimated spectrophotometrically (Roche, Germany). The study design was approved by the Animal Users and Care Committee of the Faculty of Medicine, Universiti Teknologi MARA. Statistical analysis was performed using one-way ANOVA and Tukey post hoc analysis. A *P* < 0.05 was considered statistically significant.

## 3. Results

No significant differences were seen in water intake and body weight between the groups (Tables 1 and 2). Water intake on day 0 however was slightly lower in LD1-60 and LD1-120 when compared to that in NSP on day 0 (*P* < 0.01).

**Figure 1:**
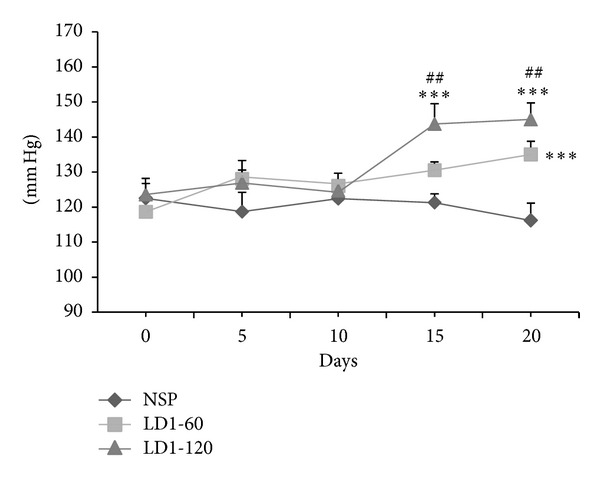
Systolic blood pressure in control and leptin treated pregnant rats. ***P < 0.001 compared to NSP group. ^##^ *P* < 0.01 comparing LD1-120 group with the LD1-60 group.

No significant differences were evident in SBP on day 0 between the 3 groups (Figure 1). Compared to the NSP, SBP was significantly higher on days 15 (LD1-120; *P* < 0.001) and 20 (LD1-60 and LD1-120; *P* < 0.001) of pregnancy in the two groups receiving leptin (Figure 1). SBP on day 20 was also significantly higher in animals receiving 120 *μ*g of leptin when compared to that in animals receiving 60 *μ*g of leptin (LD1-60 versus LD1-120; *P* < 0.01).

**Figure 2:**
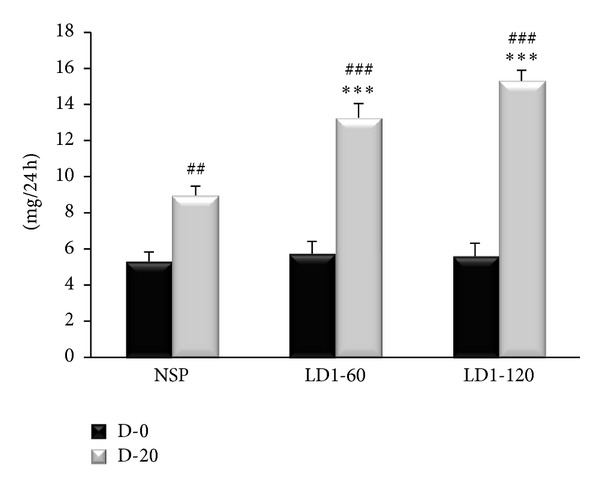
Urinary protein excretion on days 0 and 20 of pregnancy in control and leptin treated pregnant rats. ***P < 0.001 compared to NSP group. ^##^ *P* < 0.01; ^###^ *P* < 0.001 compared to their respective values at day 0.

No significant differences were evident in 24-h urinary protein excretion on day 0 between the three groups (Figure 2). Urinary protein excretion was significantly higher in leptin treated groups at day 20 of pregnancy when compared to that in NSP group (*P* < 0.001). No significant difference was present in 24 h urinary protein excretion between LD1-60 and LD1-120. 

**Figure 3:**
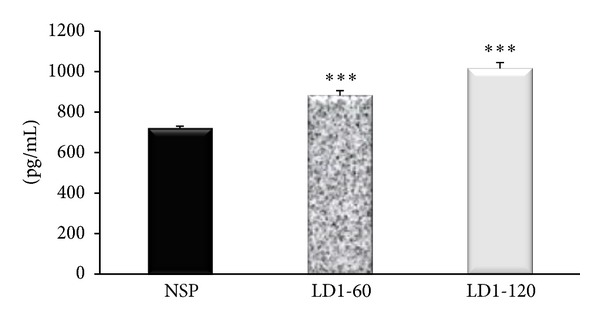
Serum concentration of ICAM-1 in control and leptin treated pregnant rats. ***P < 0.001 compared to NSP group.

Serum ICAM-1 concentrations on day 20 of pregnancy were significantly higher in the groups receiving leptin when compared to the NSP group (*P* < 0.001; Figure 3). 

**Figure 4:**
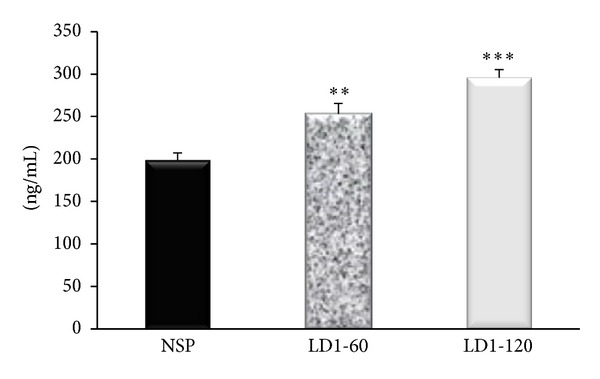
Serum concentration of E-selectin in control and leptin treated pregnant rats. **P < 0.01; ***P < 0.001 compared to NSP group.

Serum E-selectin concentrations on day 20 of pregnancy were significantly higher in the groups receiving 60 and 120 *μ*g/kg/day of leptin when compared to the NSP group (*P* < 0.01 and *P* < 0.001 resp.; Figure 4).

## 4. Discussion

The major significant findings of this study are as follows: (a) raised blood pressure during the latter part of pregnancy, (b) raised urinary protein excretion, and (c) significantly higher levels ICAM-1 and E-selectin in the serum following leptin administration during pregnancy. The precise mechanism responsible for these remains unclear. Body weight was not significantly different between the three groups either on day 0 or day 20 (Table 1). Although water intake was lower at day 0 in leptin-treated groups, there was however no significant difference in water intake between the groups on day 20 of pregnancy (Table 2). The reason for the lower water intake on day 0 might be related to incomplete or poor acclimatisation to the metabolic cages by some of the animals, as measurements were made after just 24 hours of acclimatisation. It is however unlikely that the differences in water intake at day 0 would have in any way contributed to the differences in blood pressure, proteinuria, or the markers of endothelial activation between leptin treated rats and the controls.

Increases in blood pressure following leptin administration in nonpregnant rats have been reported before [7, 23], but no reports exist of this during pregnancy. This is the first of its kind examining the effect of leptin on blood pressure and proteinuria during pregnancy. Although the mechanism responsible for the rise in blood pressure following leptin treatment was not examined in this study, increases in blood pressure following leptin administration have been attributed to increases in sympathetic activity [24, 25], increased vasomotor activity and endothelial activation [26, 27], and/or alteration in renal salt and water handling [28]. It is possible that either one of these or all of them might be involved in the raised blood pressure following leptin treatment during pregnancy (Figure 1). It was also interesting to note that increases in blood pressure following leptin treatment only became evident during the second half of the pregnancy, that is, sometime between days 10 and 15. The precise reason for this “time lag” is unclear as in our other current studies involving nonpregnant rats, blood pressure increases become evident from as early as day 7 following daily leptin treatments (unpublished data). Serum leptin levels or the time course changes in serum leptin levels were not measured in this study as we did not expect to see any significant differences in serum leptin levels between the three groups. Our previous study in the male rats did not show any significant differences in serum leptin levels between control rats and those given 30 *μ*g/kg/day of leptin for 42 days [20]. As leptin was injected as a single daily dose, and given its short half-life, it is unlikely that any significant differences in serum leptin levels would have persisted long enough to become evident between the groups, particularly when the blood was collected 24 hours after the last leptin injection. Moreover the change in body weight over the course of pregnancy was not different between the controls and leptin treated rats. Serum leptin levels however generally increase during pregnancy due to the increase in adipose tissue and we expect a somewhat similar increase in serum leptin in all the groups. Leptin injections would have just caused a transient increase in serum leptin levels, which was probably responsible for the noticeable differences in some of the measured parameters between the groups. SBP did not increase in nonleptin treated rats, suggesting that the increases in serum leptin concentrations during pregnancy do not significantly alter blood pressure. This might be due to concurrent vasodilatory effects of progesterone, as its levels increase during pregnancy. The increasing levels of progesterone during the early part of pregnancy could also explain the “time lag” between the start of leptin treatment and the increase in blood pressure. Interestingly, the effect of leptin on food intake was absent, as evident from similar increases in body weight in control and leptin treated rats, despite the raised serum leptin levels during pregnancy and this is believed to be due to concurrent leptin resistance that develops during pregnancy.

While the exact link between raised blood pressure and markers of endothelial activation is not evident from this study, the finding of elevated levels of ICAM-1 and E-selectin (Figures 3 and 4) might suggest a concurrent leptin-induced alteration in the release of vasoactive substances, like ET-1, which might be responsible for the raised blood pressure. Raised serum levels of E-selectin have been reported in cases of essential hypertension but the mechanism of the link between the raised blood pressure and increased levels of E-selectin remains unclear [29]. There are however no data available in the literature on the impact of leptin on markers of endothelial activation during pregnancy, despite the proposed link between adiposity and endothelial dysfunction [30–32]. A recent study *in vitro* however reported that leptin increased ICAM-1 production by HUVEC in a dose-dependent manner *via* the mitogen-activated protein kinase pathway [33] and a similar mechanism might also be responsible for the increases in ICAM-1 and E-selectin levels in this study. Some previous studies in nonpregnant animals have also linked leptin to induction of endothelial dysfunction secondary to oxidative stress [34], decreases in paraoxonase activity, platelet aggregation, migration, hypertrophy, and proliferation of vascular smooth muscle cells [35]. Measurement of vasoactive factors of endothelial origin like ET-1, ACE I, or eNOS activity could provide a clearer picture of the actions of leptin besides its action on the markers of endothelial activation. 

The precise mechanism responsible for the increased urinary protein excretion in leptin treated rats in this study is unclear (Figure 2). Little exists in the literature on the effect of leptin on renal protein handling. It is however known that leptin is mainly excreted by the kidney, and that rat and mouse kidneys show abundant Ob-R transcripts, indicating an interaction of leptin with special renal receptors [36, 37]. Long-term infusion of leptin in obese, diabetic db/db mouse however, has been found to induce glomerular expression and accumulation of type IV collagen [38]. Raised deposition of collagen and lesion to endothelial cells might lead to proteinuria [39]. Renal disease has been associated with obese individuals, and increased TGF-*β* and glomerulosclerosis have been reported in patients with high circulating leptin levels [39, 40]. Besides, TGF-*β* is also known to promote renal fibrosis through stimulation of extracellular matrix proteins [41]. These could be responsible for the raised protein excretion in leptin treated rats. Clearly, microscopic examination of the kidney could help provide some clues to the possible mechanism of leptin induced proteinuria. 

From the results of this study it seems that leptin administration to normotensive *Sprague Dawley rats* during pregnancy significantly increases systemic blood pressure and markers of endothelial activation. In addition, it also increases urinary protein excretion. There is therefore a possibility that raised leptin levels might contribute to some of the symptoms of pre-eclampsia. However, further studies including the measurement of vasoactive substances and renal histopathology with TGF expression are required to examine the underlying mechanism of the raised BP and increased protein excretion following leptin treatment. The findings of this study nevertheless do point to a potential role for leptin in pregnancy-induced hypertensive disorders. 

## Figures and Tables

**Table 1:** Body weight on days 0 and 20 of pregnancy in NSP, LD1-60, and LD1-120 groups.

Group	Body weight (gm)	
	Day 0	Day 20
NSP	198 ± 3.6	295 ± 3.4^ ###^
LD1-60	198 ± 3.9	316 ± 10.3^###^
LD1-120	209 ± 1.8	302 ± 6.6^###^

Values are expressed as mean ± S.E.M.  (*n* = 8 per group).

^###^ *P* < 0.001  compared to their respective values at day 0.

**Table 2:** Water intake on days 0 and 20 of pregnancy in NSP, LD1-60, and LD1-120 groups.

Group	Water intake (mL/24 h)	
	Day 0	Day 20
NSP	18.4 ± 4.44	22.4 ± 4.24
LD1-60	11.0 ± 1.56**	19.1 ± 1.1^#^
LD1-120	12.3 ± 1.81**	25.1 ± 3.43^##^

Values are expressed as mean ± S.E.M. (*n* = 8 per group).

**P < 0.01 compared to NSP group.

^#^ *P* < 0.05; ^##^ *P* < 0.01  compared to their respective values at day 0.
